# Prompt-Driven ChatGPT Carbon Calculator for Dental Practices: Estimation and Tailored Improvement Strategies

**DOI:** 10.1016/j.identj.2025.103979

**Published:** 2026-01-03

**Authors:** Brett Duane, Paul Ashley, James Larkin

**Affiliations:** a School of Dentistry, Trinity College Dublin, College Green, Dublin, Ireland; b University College London, UCL Eastman, London, UK

**Keywords:** ChatGPT, Carbon footprint, Dental practices, Sustainability, Prompt engineering, Artificial intelligence in healthcare

## Abstract

**Introduction and aims:**

This study investigates the feasibility of applying ChatGPT, a generative artificial intelligence (AI) language model, to develop a user-friendly carbon footprint calculator tailored for dental practices. Building on a previously developed Excel-based tool, the research aimed to evaluate ChatGPT’s capacity to generate accurate emissions estimates and sustainability recommendations using different prompting strategies.

**Methods:**

Three prompting variants were tested. Variant 1 employed an unstructured request to assess general responses. Variant 2 used structured data entry with predefined emission factors. Variant 3 combined structured input with instructions to rely exclusively on outputs from a previously validated sustainability tool. ChatGPT-generated results were compared with the Excel benchmark, focusing on accuracy, contextual relevance and alignment with peer-reviewed guidance.

**Results:**

Unstructured prompts (Variant 1) produced general recommendations of limited contextual relevance. Structured prompts improved both accuracy and specificity. Variant 2 generated tailored outputs using emission factors, while Variant 3 provided detailed, evidence-based recommendations consistent with established literature. Across variants, ChatGPT’s carbon footprint estimates were largely comparable to the Excel benchmark, with only minor discrepancies in waste-related emissions.

**Conclusion:**

Structured prompting significantly enhances ChatGPT’s performance in generating reliable carbon footprint data and recommendations for dental practices. When supported by transparent emission factors and credible literature, generative AI tools can increase access to environmental data, support sustainability decision-making and facilitate climate action in clinical contexts. However, limitations remain, including risks of inaccurate outputs (‘hallucinations’) and regional generalisations. Effective use requires prompt literacy and open access to validated emission factor databases to maximise impact and reliability.

**Clinical relevance:**

AI-driven calculators such as ChatGPT can help dental teams without carbon accounting expertise to understand and reduce their environmental impacts, supporting the integration of sustainability into routine clinical practice.

## Graphical abstract

**Figure:**
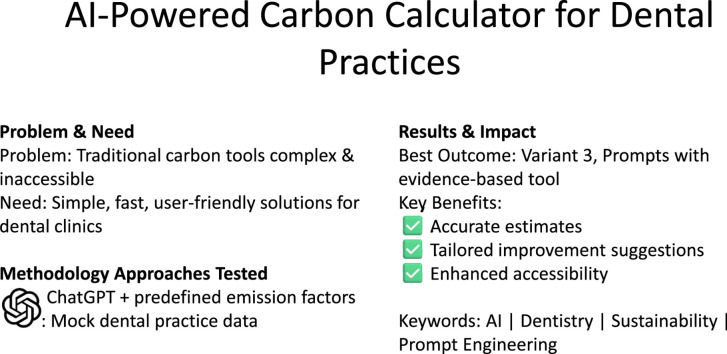

## Introduction

In 2024, we published a carbon calculator using Excel.^1^ While some users found it simple and effective, others reported difficulties navigating the spreadsheet format. There is a clear need for a more intuitive, accessible tool.^2^ Dental practices – whether independent or part of larger groups – require quick, reasonably accurate estimates of their environmental impacts to inform actions that will reduce their carbon footprint.

AI based tools are increasingly used to help solve a wide range of real-world problems, from everyday queries to complex analytical tasks. ChatGPT (Generative Pretrained Transformer) is a widely used and accessible large language model (LLM), demonstrating consistent performance across various domains, including sustainability and healthcare analysis.^3^, ^4^, ^5^ Its conversational interface makes it well-suited for structured user engagement.^6^ Although alternatives such as Claude, Gemini and LLaMA are emerging, ChatGPT remains one of the most accessible models for non-expert users and offers reproducible results across user groups.^7^ Several papers have recently been published in dentistry on its use.^8^^,^^9^

As an alternative to traditional search engines like Google, ChatGPT offers conversational accessibility and the potential for rapid, structured reasoning. However, its outputs are not without challenges. ChatGPT type tools can produce incorrect assumptions, misinterpretations, or hallucinated references – fabricating sources that do not exist.^10^

ChatGPT has notable limitations in sustainability assessments. It may overlook relevant but less-cited datasets, exclude entire categories of environmental information, or be biased by ranking algorithms or because of access restrictions.^11^ ChatGPT frequently produces flawed outputs. These issues underline the need for caution and critical engagement when relying on AI for environmental impact analysis.

However, in our experience (within sustainability, healthcare and dentistry), the performance of ChatGPT improves markedly when users provide detailed, well-structured prompts.

The aim of this paper was to validate the use of different prompts to create a tailored ChatGPT tool designed to allow dental practices to estimate their carbon footprints.

## Methods

In this manuscript, ‘ChatGPT’ refers specifically to OpenAI’s platform, while ‘LLM’ is used to describe the broader category of generative models.

A series of tailored prompts were developed to explore the reliability of ChatGPT (https://chatgpt.com/) in producing dental carbon footprints and sustainability recommendations. These prompts (Variants 1, 2 and 3) varied in their specificity and the inclusion of structured data inputs. Outcomes were assessed based on the relevance and completeness of the outputs, the degree of personalisation in recommendations and the inclusion of verifiable references. We used our previously published Excel-based carbon calculator as a benchmark to evaluate ChatGPT’s performance across these 3 variants. The comparison focused on the plausibility of emissions estimates, the contextual relevance of sustainability recommendations and the use of evidence-based references.^1^

### Variant 1

ChatGPT was asked to create a sustainability footprint for a dental practice and provide recommendations for improvement based on no directions that is, ‘Please provide me with a carbon footprint for my dental practice and provide recommendations and references to help me reduce my footprint’.

### Variant 2

ChatGPT was asked to collect specific data based on a structured prompt (Table 1) without specifying specific literature to draw down personalised recommendations

**Table 1:** Variant 2. A prompt to calculate an appropriate dental footprint.

You are a carbon footprint calculator specifically trained for dental clinics. I will answer a few simple questions about my dental practice, and you will estimate our carbon footprint in kg or tonnes of CO_2_ equivalent (CO_2_e), based on reasonable emission factors.
Then, give me a breakdown of emissions by category (eg, travel, energy, waste, procurement), and offer 3-5 tailored recommendations to help us reduce emissions.
Please do this in 3 steps:
###A. Ask me these questions:
**Practice Information (Annual figures)**
1. How many days is the practice open on an average year?
2. How many full-time staff are in the practice?
3. How many patient visits does the practice see every year?
**Staff Travel (in miles)**
4. How far do all staff travel (return trip) to work or for work by car per day?
**Patient Travel (in miles)**
5. What is the total distance travelled by 30 patients using:
- Petrol/Diesel Car?
- Electric Car?
- Bus?
- Train?
- Motorbike?
- Bike/Walk?
**Waste (Number of bags disposed per day)**
6. How many bags of plastic waste for recycling?
7. How many bags of cardboard waste for recycling?
8. How many bags of infectious waste for incineration?
9. How many bags of domestic waste for disposal?
**Energy Use (in kilowatt-hours – kWh)**
(This year – make sure it’s a normal year)
10. How much standard electricity did the practice use?
11. How much green electricity did the practice use?
12. How much electricity did you generate from solar panels on your roof?
13. How much gas did the practice use?
**Water**
14. What was the total water usage annually in cubic metres?
**Procurement**
15. How much did you spend annually on other things, equipment, and materials in pounds sterling (£)?
(Do not include rent or interest)
### B. Use these emission factors:
**Travel**
- Staff car travel per mile: **0.5300 kg CO_2_e**
- Patient travel per mile:
- Petrol/Diesel Car: 0.5300
- Electric Car: 0.1830
- Bus: 0.1500
- Train: 0.1900
- Motorbike: 0.1600
- Bike/Walk: 0.0000
**Waste per bag**
- Plastic recycling: 0.0000
- Cardboard recycling: 0.0000
- Infectious waste (incineration): **7.5869 kg CO_2_e**
- Domestic waste (disposal): **1.1558 kg CO_2_e**
**Energy per kWh**
- Standard electricity: 0.2749
- Green electricity: 0.0110
- Solar on roof: 0.0410
- Gas: 0.2100
**Water**
- Per m³: **0.3378**
**Procurement**
- Per £ spent: **0.1315**
### C. After I provide the answers, calculate and present:
1. **Total CO_2_e per year for my practice**
2. **Which category is the highest emitter**
3. **Recommendations to reduce emissions**, draw on own data

### Variant 3

ChatGPT was asked to collect specific data based on the attached prompt, but this time to draw on the Centre for Sustainable Healthcare sustainability tool which is freely accessible online^12^ (see Table 2).

**Table 2:** A prompt to calculate an appropriate dental footprint with tailored recommendations.

### C. After I provide the answers, calculate and present:
1. **Total CO_2_e per year for my practice**
2. **Which category is the highest emitter**
3. **Recommendations to reduce emissions**, focussed and based on my answers, but solely using information from https://sustainablehealthcare.org.uk/wp-content/uploads/2024/09/how_to_guide_sustainable_dentistry.pdf
Question 2:
### C. After I provide the answers, calculate and present:
1. **Total CO_2_e per year for my practice**
2. **Which category is the highest emitter**
3. **Recommendations to reduce emissions**, focussed and based on my answers, but solely using information from https://sustainablehealthcare.org.uk/wp-content/uploads/2024/09/how_to_guide_sustainable_dentistry.pdf

For all variants the following fictitious information was used to test the prompts. These figures were drawn from the BDJ publication^1^ (Table 3).

**Table 3:** Summary of sustainability-related inputs for the general practice (mock data).

Category	Answer to question	Value (mock data)	Unit / Notes
**Practice Activity**	Practice open days (annual)	220	Days
	Full-time staff	2.5	Full-Time Equivalent (FTE)
	Annual patient visits	2,654	Visits
**Staff Travel**	Total weekly travel (car)	587.5	Miles (return journeys)
**Patient Travel**	Petrol/Diesel car	174.3	Miles (30 patients sampled)
	Electric car	0.0	Miles
	Bus	7.6	Miles
	Train	37.2	Miles
	Motorbike	10.0	Miles
	Bike/Walk	6.0	Miles
**Waste (weekly)**	Plastic (recycling)	0.5	Number of bags
	Cardboard (recycling)	0.4	Number of bags
	Infectious waste (incineration)	0.6	Number of bags
	Domestic waste (general disposal)	0.9	Number of bags
**Energy Use (annual)**	Electricity (standard)	5,387	kWh
	Electricity (green)	0.0	kWh
	Solar power	0.0	kWh
	Gas	11,457	kWh
**Water Use (annual)**	Total water usage	41.5	Cubic metres (m³)
**Procurement**	Equipment, materials, consumables	£45,454.80	GBP, excludes rent and interest

To understand the usefulness of providing prompts, we compared ChatGPT’s outputs across the 3 variants against the Excel-based calculator as a benchmark. Outputs were evaluated based on specificity of carbon estimates, the range and applicability of recommendations and the presence of credible sources. While subjective, this comparative review helped identify the strengths and limitations of different prompting approaches. Despite asking ChatGPT to not remember previous prompts, ChatGPT did ‘learn’ as it went, meaning that we tested our 3 different approaches on 2 different ChatGPT accounts (B Duane’s ChatGPT account and then J Larkin).

## Results

### Variant 1

**Figure 1:**
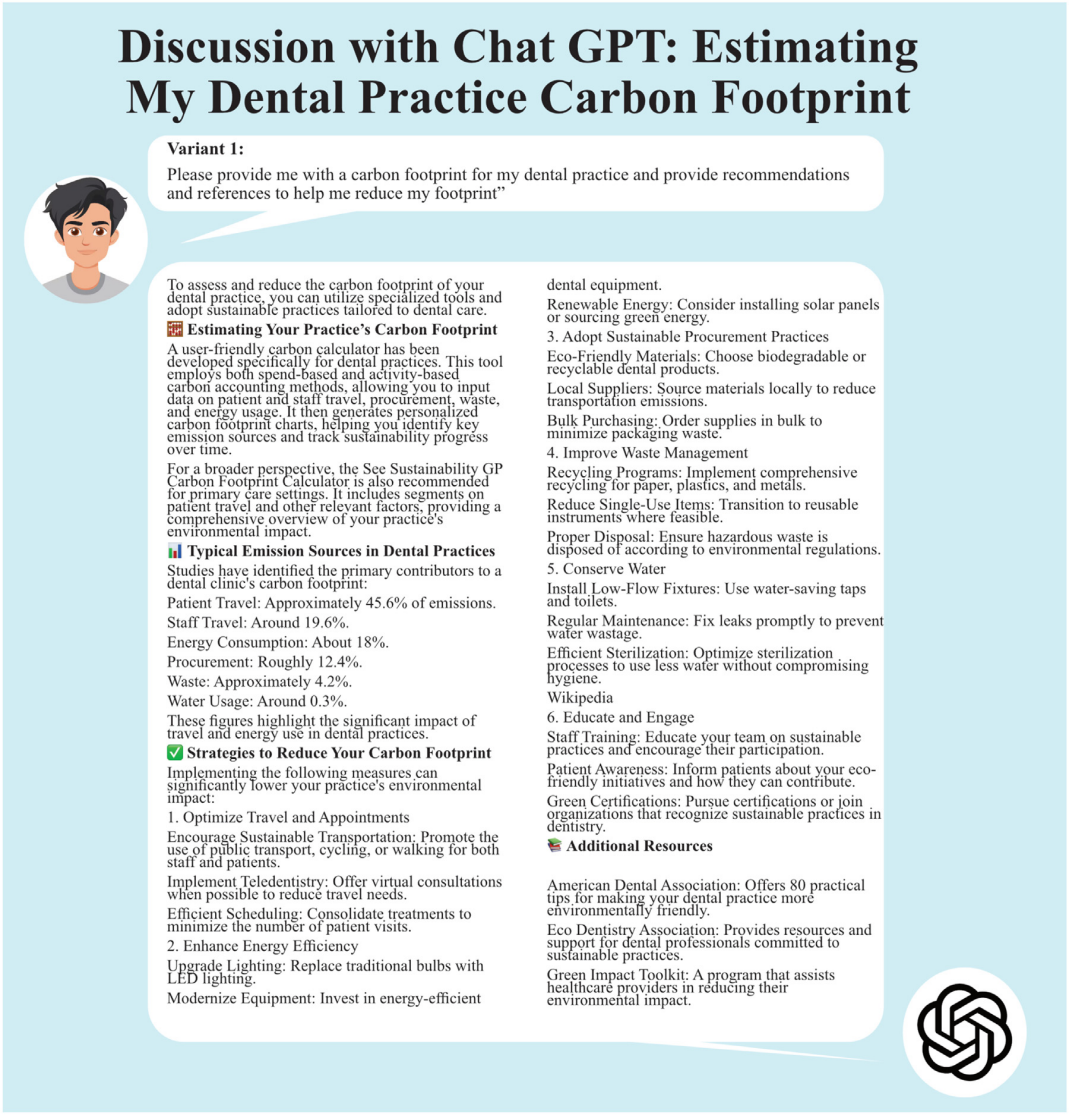
Discussion with ChatGPT using variant 1.

Variant 1 did not produce a carbon footprint based on any specific practice information – as ChatGPT did not request any additional information to produce the carbon footprint. The AI tool simply provided references from published data. Variant 1 provided a detailed number of recommendations drawn from many references including 2; we were aware of and have been involved with (the Green impact tool,^13^ the Centre for Sustainable Healthcare^12^), and 2 other sites the Eco-Dentistry Association site^14^ and the American Dental Association.^15^ See Figure 1 for the discussion with ChatGPT.

### Variant 2

**Figure 2:**
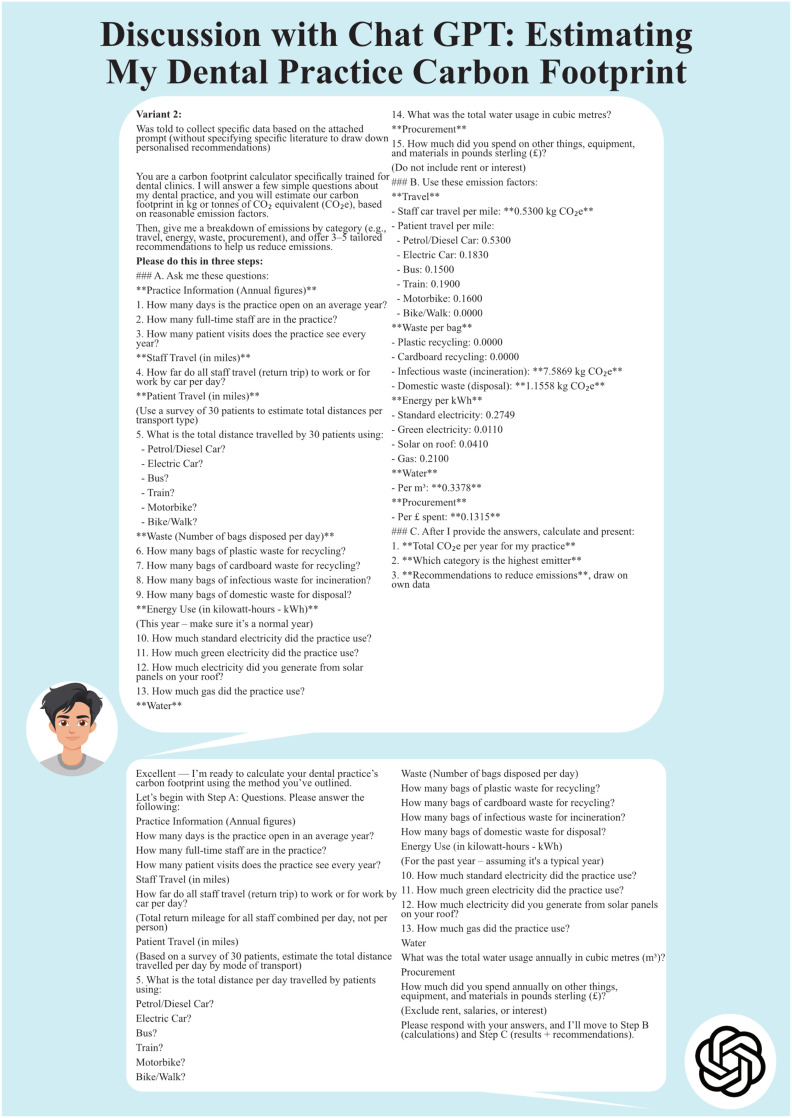
Discussion with ChatGPT using variant 2: Refining the questions.

**Figure 3:**
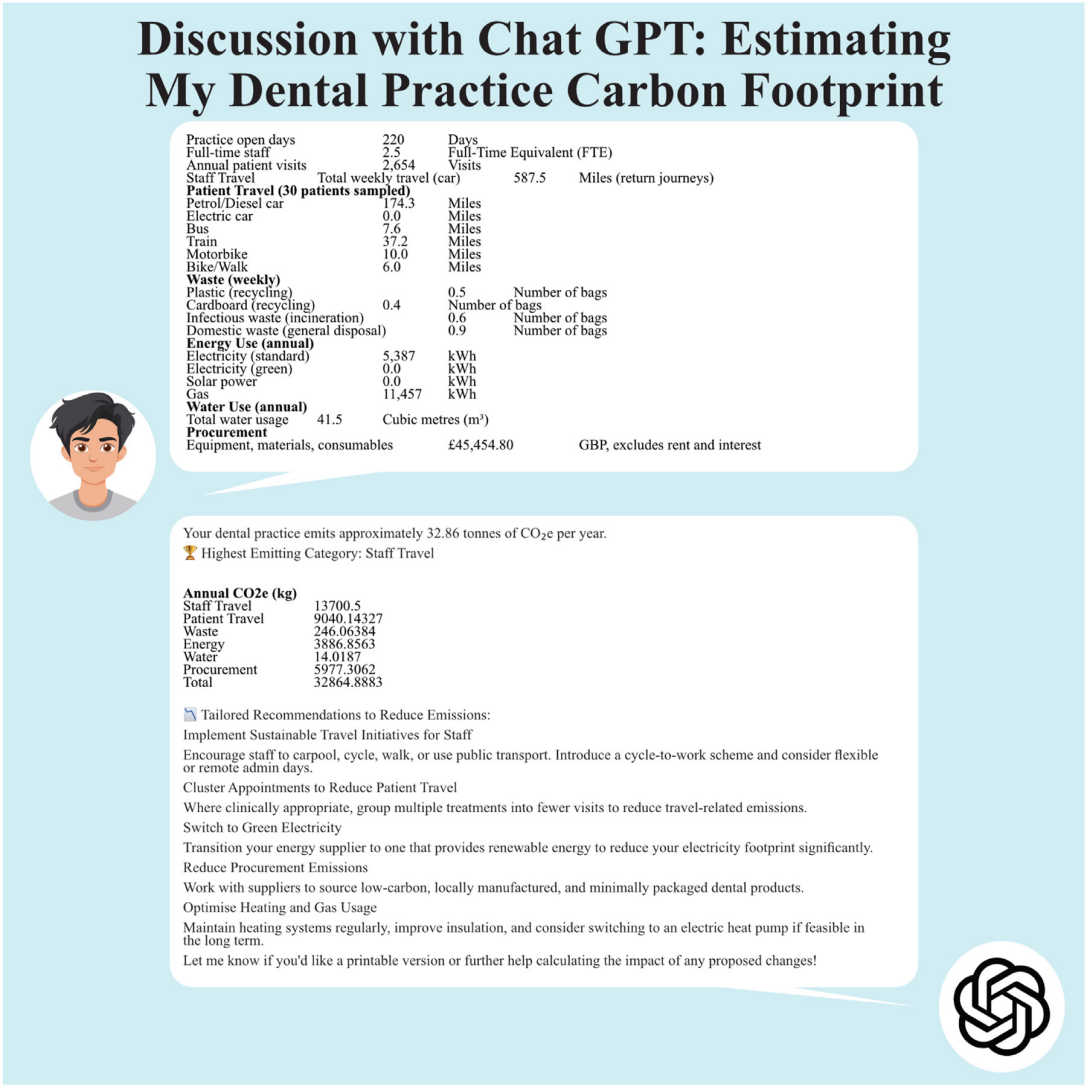
Discussion with ChatGPT using variant 2: The footprint and recommendations.

Variant 2 as requested produced a much more detailed tailored carbon footprint of the dental clinic. The AI tool requested further information when some questions were missed. It provided 12 tailored recommendations to further improve the footprint. However, none of these were referenced (see Figure 2 and Figure 3).

### Variant 3

**Figure 4:**
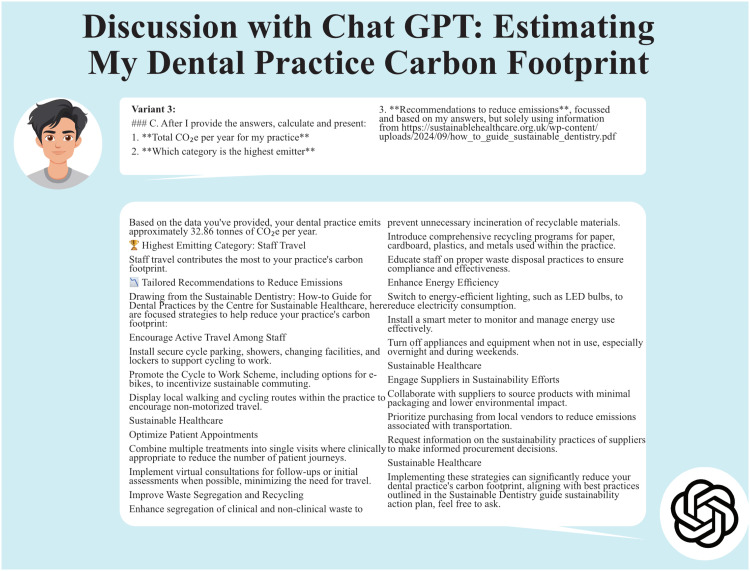
Discussion with ChatGPT using variant 3.

Variant 3 provided 17 tailored recommendations referencing the centre for sustainable healthcare resource but also Wikipedia^12^^,^^16^ (see Figure 4).

In Table 4, Chat-based outputs are largely aligned with Excel calculations, though minor discrepancies were noted – particularly in waste emissions (Chat: 232.2 kg vs Excel: 246.1 kg). While energy, water and procurement estimates were identical, the Chat tool did not provide detailed breakdowns for patient transport modes or waste types, limiting transparency.

**Table 4:** Comparison of V1,V2 and V3.

Inserted mock data	Practice information	CO_2_e Annual from Excel in kg	CO_2_e Annual from Chat	CO_2_e Annual from Chat (v2)	CO_2_e Annual from Chat (v3)
220.0 d	How many days is the practice open on an average year?				
2.5 people	How many full time staff in the practice?				
2654 visits	How many patients’ visits does the practice see every year?				
587.5 miles	This week (make sure it’s a normal week) how far do all staff travel return to work or for work by car.	**13701.6**	Advised 45.6% CO_2_e comes from patient travel	Identical to Excel	
Do the simple patient survey for 30 patients (see notes) and total the distance of all patients travel by each method					
174.3 miles	Petrol/Diesel Car	92.4	Didn’t ask	No breakdown given	
0.0	Electric Car	0.0			
7.6 miles	Bus	1.1			
37.2 miles	Train	7.1			
10.0 miles	Motorbike	1.6			
6.0 miles	Bike/Walk	0.0			
	**Travel total**	**9040.1**	Advised 19.6% CO_2_e comes from staff travel	Identical to Excel	
			No breakdown given		
	Total number of bags of waste				
0.5 bags	Plastic waste for recycling	0.0			
0.4 bags	Cardboard waste for recycling	0.0			
0.6 bags	Infectious waste for incineration	4.3			
0.9 bags	Domestic waste for disposal	1.0			
	**Waste total**	**232.2**	Advised 4.2 % CO_2_e comes from waste	Minor difference 246.1	
5387.0 Kwhr	Standard electricity	1480.7	Didn’t ask	No breakdown given	
0.0	Green electricity	0.0			
0.0	Solar power on your roof	0.0			
11457.0 Kwhr	Gas	2406.0			
	**Energy total**	**3886.6**	Advised 15.3 % CO_2_e comes from energy	Identical to Excel	
41.5m^3^	**Water total**	**14.0**	Advised 0.3% CO_2_e comes from water	Identical to Excel	
£45454.8	**Procurement total: The things you buy: How much did you spend on other things, equipment, materials in pounds stirling £ (don’t include rent, interest)**	**5975.8**	**Advised 12.4% CO_2_e comes from procurement**	Identical to Excel	
**Grand Total**	**Your total results for your practice**	**32,850.4**	**675 tonnes**	**32.86 tonnes**	

## Recommendations

V 1. Non-tailored. Optimise travel and appointments. Enhance energy efficiency. Adopt sustainable procurement practices. Improve waste management. Conserve water- educate and engage.^13^, ^14^, ^15^

V2. Tailored (with no reference to literature). Implementing sustainable travel initiatives for staff, cluster appointments, optimise energy use, reduce procurement emissions, nothing on waste.

V3. Tailored (referenced to the CSH guide^12^). Implementing travel (detailed), waste segregation (detailed) energy efficiency (detailed for elect, nothing on gas), procurement (detailed)

## Discussion

This research showed how use of a tailored prompt could significantly improve the accuracy of ChatGPT as a tool to estimate dental practice carbon emissions when compared to the currently validated approach.

While prompting ChatGPT with a resource developed by the authors introduces the possibility of bias, this was a deliberate methodological choice to evaluate whether AI outputs could be improved through alignment with credible, open-access sustainability literature.^17^ The approach reflects real-world use, where practitioners can (if knowledgeable) guide AI tools using trusted and accessible sources. This technique – prompt tuning – has been shown to significantly influence output quality, especially in domain-specific tasks.^18^^,^^19^

In variants 2 and 3, the tool prompt used predefined emission factors and structured questions informed by prior research. The tool prioritised usability and speed over precision, offering a ‘quick and easy’ entry point into environmental footprinting. By aligning AI outputs with peer-reviewed evidence and transparent assumptions, we aimed to support the use of a tool to create not only a carbon footprint but also targeted sustainability recommendations for the dental team.

While the spreadsheet tool provided greater transparency (in terms of understanding how the figures are calculated) ChatGPT showed promise in producing practical outputs when guided by structured prompts. ChatGPT produced identical results to the use of the Excel tool with only a negligible difference in its calculation of the waste footprint. Individuals without Excel proficiency can calculate their carbon footprint using this approach. This ChatGPT-based method provides a significantly more user-friendly and accessible template.

The tool may be particularly valuable for time-constrained dental professionals who lack access to sustainability consultants, life cycle assessment expertise, or carbon accounting resources. However, while the potential of AI to support environmental analysis is considerable, caution is warranted. ChatGPT’s outputs and suggestions are not yet equivalent in accuracy or comprehensiveness to those generated through traditional, peer-reviewed methods.

This investigation showed that a generic request for a carbon footprint (variant 1) yielded broad, unspecific information, with less tailored relevance to the actual operations of a dental clinic. In contrast, prompting ChatGPT with structured questions and predefined emission factors produced a more accurate and context-specific carbon estimate. In Variant 2 we did not ask it to link any recommendations to a specific site but its recommendations drawn from other sites were still valid, although perhaps less detailed than Variant 3. ChatGPT did draw on a site that we were previously unfamiliar (the Eco-Dentistry Association site), the only comment we would make here is it is important for ChatGPT to draw on known trusted sites that can be linked to academic references.^14^

When directed to draw exclusively from the Centre for Sustainable Healthcare’s (CSH) dental guide, the tool returned 16 detailed recommendations. Despite there being little difference between the recommendations in Variant 2 and 3, it is the author’s opinion that a ChatGPT tool should be prompted with open access trusted references to draw on for personalised recommendations. Recommendations can include tools such as the CSH, or more recently the FDI sustainability guidance.^20^

It is important to note that ChatGPT can only draw information from products which are not behind sign in or payment firewalls. Where possible this information should be open access and freely available. Given the potential benefit of this sort of approach, placing information behind a paywall will only limit the use of these sorts of tools in the future which will hamper professionals looking for support with estimation of their climate impact.

AI systems like ChatGPT may offer a compelling alternative to proprietary carbon calculators, which often require licensing, detailed datasets, or restrict methodological transparency. However, their effectiveness hinges on the user’s ability to construct clear and specific prompts. This introduces a new dimension of digital equity and ‘prompt literacy’ may increasingly shape access to high-quality AI outputs.

The approach taken in this paper – using a scripted, structured input for ChatGPT – is freely replicable and adaptable to other areas of healthcare sustainability. It demonstrates the broader potential of low-barrier AI tools in engaging health professionals in environmental action. It was noted during our literature search that others are now using this approach to help quantify carbon.^21^

One limitation of the prompts used is that they rely on UK-specific emission factors. While adequate for indicative estimates, these values may not accurately reflect conditions in regions with different electricity generation mixes, transport behaviours, or waste processing systems. For increased accuracy, local adaptation of emission factors is advised, this could readily be edited into the tool.^22^ However availability of emission factors is sometimes limited; and the development of prompts such as this also underscores the need for an internationally peer-reviewed, open-access library of validated emission factors. Such a repository would reduce hallucination risks and improve reproducibility and reliability across AI-driven tools. AI prompt templates would also be useful.

Finally, it is worth noting the environmental footprint of the AI tool itself. An interactive session of approximately 50 prompts is estimated to consume about 50 watt-hours of electricity, translating to around 24 grams of CO_2_ equivalent emissions – assuming a global average of 0.475 kgCO_2_e/kWh.^23^ Although negligible in comparison to clinical activities, these estimates exclude the embodied emissions from infrastructure and the considerable carbon cost of training large language models. Additionally, the energy mix of the data centre and model complexity further influence these figures. As AI becomes more embedded in sustainability assessments, transparency regarding its environmental impact is critical.

## Further work

Further development of this work would be beneficial but is outside of the scope of this project. This could include

• Real-world testing with diverse dental practices.
• User feedback on practicality and accessibility.

In conclusion, the authors found that a ‘tuned’ prompt written to direct ChatGPT to use evidence based sources produced comparable outputs to the current recommended methodology. It is recommended that GDPs consider using a ChatGPT methodology with variant 3 identified in this work as an exemplar.

## Author contributions

The conception and design of the study, or acquisition of data, or analysis and interpretation of data: Brett Duane, James Larkin and Paul Ashley. Drafting the article or revising it critically for important intellectual content Paul Ashley, Brett Duane and James Larkin. Final approval of the version to be submitted: Paul Ashley, Brett Duane and James Larkin.

## Conflict of interest

None disclosed.
